# Identification of different plastic types and natural materials from terrestrial environments using fluorescence lifetime imaging microscopy

**DOI:** 10.1007/s00216-024-05305-w

**Published:** 2024-04-23

**Authors:** Maximilian Wohlschläger, Martin Versen, Martin G. J. Löder, Christian Laforsch

**Affiliations:** 1 Faculty of Engineering Sciences, Rosenheim Technical University of Applied Sciences, Hochschulstraße 1, 83024 Rosenheim, Germany; 2 Animal Ecology I and BayCEER, University Bayreuth, Universitätsstraße 30, 95440 Bayreuth, Germany

**Keywords:** Plastic identification, Microplastic contamination, Terrestrial pollution, Microplastic in soils, Fluorescence lifetime, FD-FLIM

## Abstract

Environmental pollution by plastics is a global issue of increasing concern. However, microplastic analysis in complex environmental matrices, such as soil samples, remains an analytical challenge. Destructive mass-based methods for microplastic analysis do not determine plastics’ shape and size, which are essential parameters for reliable ecological risk assessment. By contrast, nondestructive particle-based methods produce such data but require elaborate, time-consuming sample preparation. Thus, time-efficient and reliable methods for microplastic analysis are needed. The present study explored the potential of frequency-domain fluorescence lifetime imaging microscopy (FD-FLIM) for rapidly and reliably identifying as well as differentiating plastics and natural materials from terrestrial environments. We investigated the fluorescence spectra of ten natural materials from terrestrial environments, tire wear particles, and eleven different transparent plastic granulates <5 mm to determine the optimal excitation wavelength for identification and differentiation via FD-FLIM under laboratory conditions. Our comparison of different excitation wavelengths showed that 445 nm excitation exhibited the highest fluorescence intensities. 445 nm excitation was also superior for identifying plastic types and distinguishing them from natural materials from terrestrial environments with a high probability using FD-FLIM. We could demonstrate that FD-FLIM analysis has the potential to contribute to a streamlined and time-efficient direct analysis of microplastic contamination. However, further investigations on size-, shape-, color-, and material-type detection limitations are necessary to evaluate if the direct identification of terrestrial environmental samples of relatively low complexity, such as a surface inspection soil, is possible.

**Graphical Abstract:**

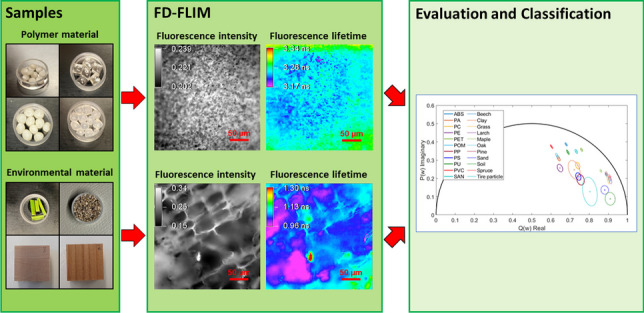

**Supplementary Information:**

The online version contains supplementary material available at 10.1007/s00216-024-05305-w.

## Introduction

Worldwide, pollution by micro- and nanoplastics (MPs and NPs) is an increasing environmental issue and burden on society [1]. The uncontrolled discharge of millions of tons of plastic waste into our environment every year [2, 3] severely endangers the world’s terrestrial and aquatic ecosystems [4–10] and poses a potential risk of unknown severity to human health [11]. The effects of MPs on organisms and different ecosystems depend mainly on the properties of the plastic particles and the level of exposure [11]. Thus, accurate, fast, and reliable determination of the number, plastic type, shape, size, and morphology is essential for assessing the contamination level and ensuring reliable ecological risk assessment.

Chemical methods are indispensable for the reliable identification of MPs. Here, two major analytical approaches have prevailed: mass-based and particle-based chemical identification methods. To determine the mass-related proportion of MP particles in an environmental sample, thermal extraction and desorption-gas chromatography/mass spectroscopy (TED-GC/MS) [12, 13] or pyrolysis GC/MS [14, 15] are used. However, TED-GC/MS or pyrolysis GC/MS methods do not provide information about the exact number, size, shape, and morphology of MP particles in an environmental sample, i.e., essential parameters for hazard assessment are not available. Alternative particle-based analytical techniques, such as Raman or Fourier transform infrared (FTIR) spectroscopy, are necessary to obtain this information on particle properties.

Meanwhile, it is clear that soil environments, especially agricultural soils, are among the most common MP-contaminated habitats [8]. However, the microplastics (MPs) analysis in soil samples still represents an analytical challenge, as soil is among the most complex environmental matrices. In addition to mass-based methods, particle-based analysis of MPs in soil samples involves time-consuming sampling, sample extraction and purification, and sample identification and quantification [8] to obtain reliable data via Raman or micro-FTIR and attenuated total reflectance (ATR) [16]. Thus, none of these spectroscopic methods is suitable for directly identifying MPs in environmental samples [17]. Generally, this hampers the fast, high-throughput analysis of MP in environmental soil samples.

To circumvent these issues, a direct and rapid method for detecting and identifying MPs in a complex environmental matrix would yield substantial efficiencies in terms of time and resource allocation. This approach would significantly increase the potential sample throughput, especially concerning MP monitoring programs. However, such methods are currently unavailable for aquatic and terrestrial environments.

Recent studies highlighted the doping of plastics with fluorescent markers to identify them using their fluorescence spectra [18, 19]. However, this approach can only be applied to study the fate of MP in the environment or for specific recycling processes, but not for identifying the numerous MP particles already present. Further, doping with fluorescent markers requires a careful and time-consuming pre-treatment of the samples and could also be toxic to our environment, depending on the substance. Nevertheless, the characteristic autofluorescence, particularly the fluorescence lifetime of plastic types, can be used as a “fingerprint” to identify polymer types [20]. At first glance, identifying plastic types utilizing their fluorescence behavior is surprising as ideal plastic types without low-lying chromophores like polyethylene (PE) or polypropylene (PP) should not have a fluorescence emission. However, technical polymers, regardless of industrial or consumer grade, have chain breaks and contain oxidation products like carbonyls from their manufacturing process, causing a fluorescence emission if excited by an intense laser pulse [21–23]. Additionally, common additives, such as optical whiteners or UV blockers, can cause fluorescence emission. From these studies, we deduce that plastics, which a priori should not exhibit fluorescence emission, nevertheless show fluorescence due to the defects mentioned and the potential presence of additives, as these plastics are generally not available in pure form due to their versatile fields of application. Therefore, plastics, such as PP and PE, which have been improperly disposed of in the environment, should also exhibit fluorescence. Time domain or frequency domain fluorescence lifetime imaging microscopy (TD-FLIM; FD-FLIM) is a promising tool for sensitive, fast, and direct identification of MPs in an environmental matrix. TD fluorescence lifetime measurements have been successfully used to distinguish between several plastic types [20, 24] and aquatic environmental materials [23]. However, whether natural materials from the terrestrial environment can also be distinguished from different plastic types is still being determined. A drawback of TD fluorescence lifetime measurements is that it requires a long measurement time and that TD measurements are performed only at a single fluorescence wavelength, which implies that only one emission state is considered when measuring the fluorescence lifetime. This single-state emission may be sound if it reveals a fingerprint of the plastic or disadvantageous if the plastic exhibits a low fluorescence intensity, thus leading to the mentioned long photon integration time.

Besides measuring fluorescence lifetimes in the TD using TD-FLIM, the lifetime can also be determined in FD [25]. It has been previously demonstrated [26] that the size, shape, and morphology of novel reference plastic particles can be determined at least down to 125 µm [27] via the high imaging quality of FD-FLIM. Additionally, a Gaussian analysis in combination with a threshold algorithm showed that MP particles can be identified on soil down to a size of 70 µm [28]. Our recent investigations [29–32] using FD-FLIM showed the high potential of this technique for plastic identification; however, it covered only three plastic types and two non-plastic materials investigated using FD-FLIM with only one excitation wavelength, whereby it is not known whether the chosen wavelength was superior to other excitation wavelengths.

Although FD-FLIM theoretically showed basic potential for identifying MPs, an optimized methodology for discriminating various polymer types from different natural materials has yet to be reported, which is an indispensable basis for identifying MPs directly in environmental samples such as agricultural soil samples. Moreover, using TD or FD fluorescence lifetime measurements, natural materials from terrestrial ecosystems have yet to be investigated.

To fill this knowledge gap and investigate the potential of FD-FLIM for discriminating a variety of plastic types from a variety of natural materials present in terrestrial environmental samples, we conducted two extensive experiments on eleven different polymer types, tire wear, and ten natural materials from terrestrial environments, using fluorescence spectroscopy and FD-FLIM analysis. We aimed to answer two primary research questions: (I) is there a general optimum wavelength for identifying different plastic types simultaneously, and (II) can plastic types and natural materials from terrestrial environments be identified and differentiated at one superior wavelength? To address the first question, we tested three different excitation wavelengths, 405 nm, 445 nm, and 488 nm, to measure the fluorescence spectra and lifetimes of the plastic samples. Using wavelengths above the ultraviolet (UV) region could be superior, as biological materials are strongly light absorbing in the ultraviolet range and thus may hamper any detection of the plastic types [20]. In addition, longer excitation wavelengths can result in the plastics emitting little or no fluorescence as their absorption coefficient converges toward zero. To answer the second question, we determined the fluorescence lifetime of ten natural materials from terrestrial environments and tire wear next to the plastics to examine whether all the materials could be distinguished and identified at one superior wavelength. Based on these results, we aimed to establish a basic analytical framework in which directly identifying microplastics in environmental matrices using the FD-FLIM method is possible.

## Materials and methods

A database of fluorescence properties must be developed to directly identify the plastic type, shape, and size in an environmental matrix via FD-FLIM. Therefore, a superior excitation wavelength must be identified to identify the plastic types that are best for database development. Spectral fluorescence and FD-FLIM measurements of eleven plastic types and tire wear were obtained with the described experimental setup. Additionally, FD-FLIM measurements of ten different natural materials from terrestrial environments similar to those found in agricultural land are conducted using the wavelength at which plastic types can be identified best. The measured spectral and FD fluorescence lifetime data were evaluated using the described algorithm.

### Theory

**Fig. 1:**
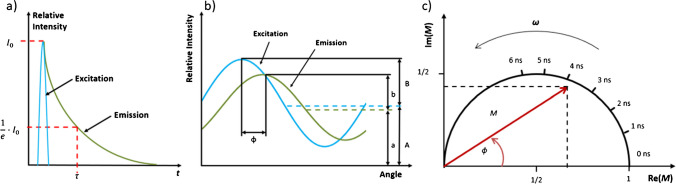
Principle of the time domain fluorescence lifetime measurements (**a**), where the fluorescence excitation is pulsed (blue) and the fluorescence emission decays exponentially (green). Method of frequency domain fluorescence measurements (**b**), where the excitation oscillation (blue) causes the harmonic, phase-shifted, amplitude-damped, and equivalent-shifted fluorescence signal (green). Schematic representation of the phasor plot (**c**), where the modulation index M is the length of the vector, which is rotated by the phase shift ϕ and displayed in a unit circle, normalized by the modulation frequency ω

The fluorescence lifetime can be determined by time domain or frequency domain fluorimetry [21]. Generally, the fluorescence lifetime *τ* is described by the time after which the maximum fluorescence intensity *I*_*0*_ decreased to *I*_*0*_*/e* (see Fig. 1a). In the time domain, the fluorescence excitation of the examined material is achieved by a laser pulse of a defined wavelength, which lasts a few nanoseconds (see Fig. 1a: blue curve). The excitation causes a Stokes-shifted fluorescence emission with a maximum intensity *I*_*0*_ at the maximum optical output power of the laser pulse. After the excitation pulse, the fluorescence intensity decays exponentially (see Fig. 1a: green line). The fluorescence lifetime in the nanosecond range is material-specific and commonly measured by a photoelectron amplifier in combination with a sensitive detector.

If the fluorescence lifetime is measured in the frequency domain, a sinusoidally or rectangularly modulated light source is used for fluorescence excitation. With this, the modulated light source has a defined modulation frequency *ω* (see Fig. 1b: blue oscillation curve). If the examined material is excited with a harmonic excitation, the fluorescence signal follows the oscillating excitation with the same frequency but is phase-shifted by the angle *ϕ*. Atomic internal conversions lower the re-emitted energy, which causes a damping in amplitude *a* and a shift in mean value *b* concerning the excitation (*B*, *A*) (see Fig. 1b: green oscillation). In frequency domain fluorimetry, two fluorescence lifetimes can be calculated by measuring the values of the phase shift *ϕ*, the amplitude damping from *A* to *a*, and the equivalent shift from *B* to *b*. Using the measured phase shift *ϕ* and the predefined modulation frequency *ω*, the phase-dependent fluorescence lifetime *τ*_*ϕ*_ can be calculated according to Eq. (1).

1 $${\tau }_{\phi}=\frac{{\text{tan}}\left(\phi \right)}{\omega }$$

The measurements of the amplitude damping (*A*, *a*) and the average shift (*B*, *b*) are used to calculate the modulation index *M* according to Eq. (2).

2 $$M=\frac{(b/a)}{(B/A)}$$

The modulation-dependent fluorescence lifetime can be derived using the modulation index *M* and defined modulation frequency *ω* by Eq. (3).

3 $${\tau }_{M}=\frac{\sqrt{\frac{1}{{M}^{2}}-1}}{\omega }$$

Furthermore, in frequency domain fluorimetry, a representation of the modulation index and phase shift is possible in a phasor plot, shown schematically in Fig. 1c. The phasor plot combines the two measured core quantities of the fluorescence decay time measurement in the frequency range: the modulation index *M*, which represents the length of the arrow starting from the origin, and the phase shift *ϕ*, which represents the rotation of *M* around the origin. In addition to plotting the modulation index *M* as the real and imaginary fraction, a quick statement about the fluorescence decay time of the medium can be made by using a half-unit circle ranging from the origin to 1 on the real parts axis. The fluorescence lifetime can be obtained directly because the half circle can be divided into segments representing the fluorescence lifetime. As a result, the half-circle segmentation depends on the modulation frequency *ω*, defined by the measurand. The higher the modulation frequency *ω* is, the lower the resolution of the measured fluorescence lifetimes.

**Fig. 2:**
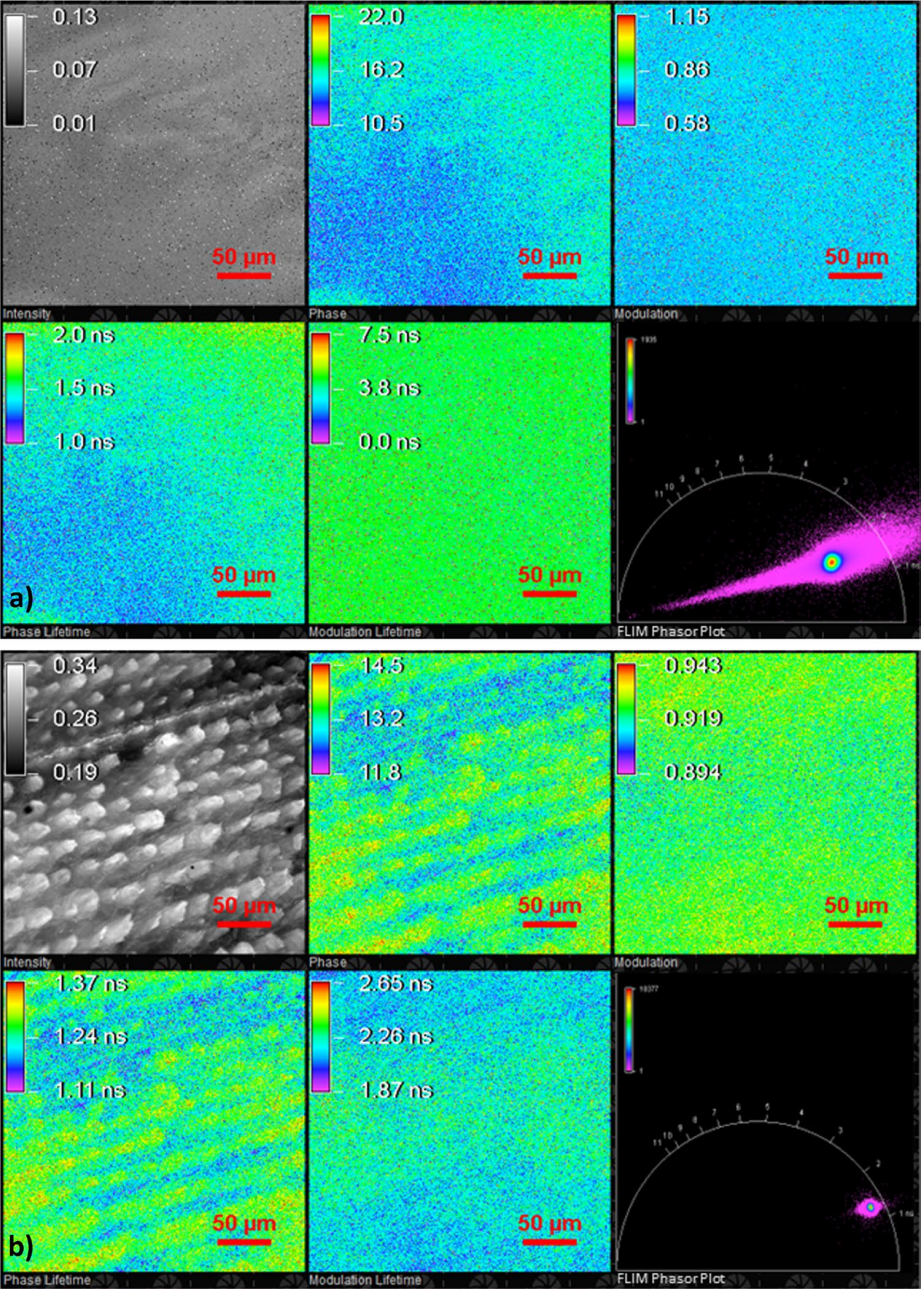
Screenshots of FD-FLIM measurements done resulting in a stack of images containing fluorescence intensity (intensity), phase-shift (phase), modulation index (modulation), phase-dependent fluorescence lifetime (phase lifetime), and modulation-dependent fluorescence lifetime (modulation lifetime) of **a** PP and **b** larch. The phasor plot of (FLIM phasor plot) is also displayed for both measurements

FD-FLIM measures the fluorescence lifetime based on the theory of frequency domain fluorimetry. As the properties of the fluorescence excitation are known, calculations of the modulation index and the phase shift, which are used to determine the phase- and modulation-dependent fluorescence lifetime, are possible. Additional information on the camera technique is given in SI section B.1. Examples of FD-FLIM measurements are shown in Fig. 2. The measurement results in an image stack of five layers: fluorescence intensity (intensity), phase shift (phase), modulation index (modulation), and phase- and modulation-dependent fluorescence lifetime (phase lifetime, modulation lifetime, respectively). In addition, the measured modulation index and phase shift can be displayed in a FLIM phasor plot, where each pixel is plotted separately with a modulation and a phase angle. As an example, the FLIM phasor plots in Fig. 2 show a distribution of values where the pixel’s color represents the pixel’s frequency with these modulation and phase values. Here, it can be seen that PP has a higher variance of values than larch (more pink values around the center) due to the lower fluorescence intensity emanating from PP. Instead of separately graphing each pixel, a FLIM phasor plot containing a mean (square) and standard deviation (ellipse) of the measured values can be extracted from the images (Fig. 4). This representation enhances the possibility of calculating the probability *P* [%] of misclassifying a measurement using Eq. (4), where ${A}_{ol}$ is the area of overlap and ${A}_{total}$ is the total area of the ellipse:

4 $$P [\%]=1-\frac{{A}_{ol}}{{A}_{total}}$$

### Experimental setup

For the investigations, an experimental setup consisting of laser diodes (LaserNest 405–300, LaserNest 445–500 and PhoxX + 488–200) from Omicron-Laserage GmbH, an FD-FLIM camera system pco.flim from Excelitas PCO GmbH, and a probe station (EPS150FA from Cascade Microtech) including a microscope (PSM-1000 from Motic) and optical filters was used (see Figure S1, SI section B2). The FD-FLIM camera can also be exchanged for a spectrometer (mini-spectrometer from Hamamatsu) to measure the fluorescence spectrum. The laser diodes have optical output powers of 300 mW (405 nm), 500 mW (445 nm), and 200 mW (488 nm). Utilizing the pco.flim, an areal measurement of 1008 × 1008 fluorescence lifetimes is possible with a dynamic range of 10 bits. Depending on the choice of magnification, a field of view of 0.28 mm × 0.28 mm (× 20 magnification), 0.56 mm × 0.56 mm (× 10 magnification), and 2.8 mm × 2.8 mm (× 2 magnification) can be achieved. Hence, taking a detector pixel size of 5.6 µm into account, a pixel resolution of 0.28 µm (× 20 magnification), 0.56 µm (× 10 magnification), and 2.8 µm (× 2 magnification) is possible. In [28], we were able to clearly identify and visualize a HDPE plastic particle with a size of 70 µm at × 2 magnification. We did not investigate the maximum spatial resolution of the used experimental setup. Transferring these results to × 20 magnification, at least particles with a size of 7 µm should be resolved clearly. Nevertheless, the actual spatial resolution has to be tested in the future. Additional information on the experimental setup can be found in [28] and SI section B2.

### Sample preparation

To determine the fluorescence properties of different plastics, eleven technical plastics (granulates) were tested. The plastic types used were acrylonitrile butadiene styrene (ABS; Novodur P2MC), polyamide (PA; Ultramid B27R, polycarbonate (PC; PANLITE L 1250Y), PE (Lupolen 6031 M), polyethylene terephthalate (PET; “Type M” Trevira), polyoxymethylene (POM; Dettin 500), PP (Moplen-HP 570 M), polystyrene (PS; BASF PS 158 k), polyurethane (PU; Desmopan 385E), polyvinylchloride (PVC; TRO/LIT VB 537-HE), and styrene acrylonitrile (SAN; Luran 368 R). According to Plastics Europe, these plastic types are chosen because they are the most frequently used base materials for application-related plastic components [1]. Five granulates are randomly picked for spectral fluorescence analysis from each plastic type, and 50 are randomly picked for FD-FLIM measurements. Random picking is performed to compensate for any dependencies of the fluorescence properties on the granules.

Eleven natural materials from terrestrial environments were collected to investigate whether the plastic types could be differentiated from a sample set of natural materials from terrestrial environments that can be found on agricultural land: grass; wood, containing the wooden types beech, maple, oak, spruce, larch, and pine; quartz sand (Quarz Sand, Quick-Mix, Germany); soil/humus (Rasenerde, Toom, Germany); and clay from a clay pot for flowers both from a German home improvement store. Additionally, tire wear material is included in these investigations because they now account for a large proportion of the microplastics in the environment [33].

### Experimental procedure for measuring the fluorescence spectra

The procedure for measuring and evaluating the fluorescence spectra was adapted and improved from [28]. To measure the fluorescence spectra of the plastic types using 405 nm excitation, the mini-spectrometer and the LaserNest 405–300 were assembled into the microscope. The corresponding pair of filters is inserted into the optical path. Additionally, the microscope’s magnification was adjusted to × 20 to measure the fluorescence spectra precisely. The optical output power is set to the maximum. An exposure time of 2 s was defined for the spectral measurement of the samples to avoid any influence of the exposure time on the spectral analysis. Once the acquisition parameters are set, a background measurement is carried out to eliminate the noise of the measurement system and the interference from the measurement environment. The measurement software automatically subtracts the measured background from the fluorescence spectra of the sample. After subtracting the background, five samples of the selected materials were placed under the microscope one after the other, and the fluorescence spectra of areas (approximately 1 mm^2^) on each sample were measured. From these five spectral measurements, an average fluorescence spectrum was calculated as described below. Upon completion of the measurements using the 405 nm laser diode, the measurements were repeated one after the other using 445 nm and 488 nm laser diodes.

The measured spectra of each excitation wavelength, plastic type, tire wear, and the terrestrial environment’s natural materials are imported into the MATLAB workspace as a data table. Next, the average of the five measured fluorescence spectra of the different materials was calculated. Then, the averaged fluorescence spectra of each material are interpolated using an interpolation step size of 0.01 nm. A local regression model using weighted linear least squares and a second-degree polynomial model smoothed the interpolated fluorescence spectra. After that, the fluorescence maximum ${I}_{{\text{max}}}$ and the corresponding wavelength ${\lambda }_{{\text{max}}}$ are obtained. The Stokes shift $\Delta \lambda$ is calculated by subtracting the excitation wavelength ${\lambda }_{Ex}$ from ${\lambda }_{{\text{max}}}$.

### Experimental procedure of an FD-FLIM measurement

Carrying out and evaluating FD-FLIM measurements was adapted and further developed from [28]. Instead of the mini-spectrometer, the FD-FLIM camera system is assembled to the microscope to measure the areal fluorescence lifetime. NIS Elements from NIKON is used to control the camera. The FD-FLIM camera must be referenced to measure the absolute values of the fluorescence lifetime. Therefore, the optical output power of the 405 nm laser diode is set to its maximum, and a reference slide from Starna Scientific with a standardized fluorescence lifetime of 3.75 ns is placed on the microscope stage. Finally, the modulation frequency is set to 30 MHz.

After referencing, the 50 granulates are placed on the sample stage, and the FD-FLIM measurements are executed. A single FD-FLIM measurement resulted in a TIF (tagged image file) stack containing five TIF images (fluorescence intensity, phase shift, modulation index, phase-dependent fluorescence lifetime, and modulation-dependent fluorescence lifetime). The captured TIF stack is saved for each of the 50 measurements for the eleven granulates, resulting in a dataset of 550 measurements. After the measurements are finished, the referencing and FD-FLIM measurements are repeated using 445 nm and 488 nm laser diodes.

The evaluation of the FD-FLIM measurements is based on a Gaussian analysis. First, 50 measured TIF stacks of each material were imported into the MATLAB workspace. Each TIF stack is split into the five channels of fluorescence intensity, phase shift, modulation index, phase-dependent fluorescence lifetime, and modulation-dependent fluorescence lifetime, resulting in 50 images per channel for each material. Next, the 50 images per channel are concatenated into one matrix of dimensions 10 × 5, i.e., a matrix of 10,040 × 5040 (1004 × 1008 pixels per image) measured values. From the concatenated matrix, histograms are created, which display the absolute frequencies of a measured value from each image channel. Gaussian curves are fitted to a histogram of absolute frequencies from each channel until an ${R}^{2}$ value of 0.95 is reached. As a result, a maximum of five fitted Gaussian curves is possible, i.e., five maxima, mean values, and standard deviations can be determined for one channel histogram. The mean value and standard deviation of the Gaussian analysis from the phase- and modulation-dependent fluorescence lifetimes ${\tau }_{\phi i}$ and ${\tau }_{Mi}$ are used for numerical identification. A graphical identification is also possible by plotting the resulting normalized Gaussian curves in a normalized frequency-fluorescence lifetime diagram. In addition, the determined means and standard deviations are plotted into a phasor plot, whereby the mean is represented as a square and the standard deviation as an ellipse. From these phasor plots, the probability *P* of misclassifying a measurement using Eq. (4) was calculated.

## Results and discussion

### Fluorescence spectroscopy

**Fig. 3:**
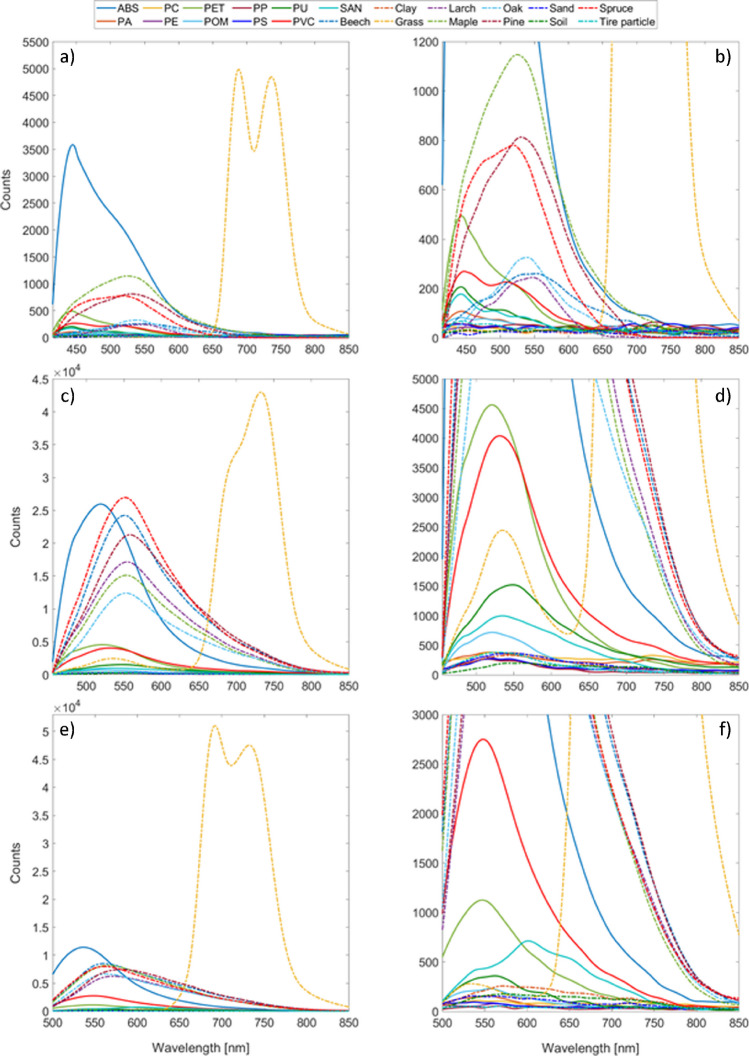
**a** Fluorescence intensity counts as a function of wavelength for the eleven different plastic types, tire wear particles, and natural materials from terrestrial environments at 405 nm excitation, whereas **b** is a detailed zoomed-in version of **a**. **c** Fluorescence intensity counts as a function of wavelength for the eleven different plastic types, tire wear particles, and natural materials from terrestrial environments at 445 nm excitation, whereas **d** is a detailed zoomed-in version of **c**. **e** Fluorescence intensity counts as a function of wavelength for the eleven different plastic types, tire wear particles, and natural materials from terrestrial environments at 488 nm excitation, whereas **f** is a detailed zoomed-in version of **e**

The resulting fluorescence spectra are shown in Fig. 3, whereby at each excitation wavelength, broad fluorescence emission in the visible is present, per the findings of Langhals et al. [20, 34]. Looking at the fluorescence spectra of the plastic types, ABS had the highest fluorescence intensity at all three excitation wavelengths. The second and third highest fluorescence intensities at 405 nm and 445 nm excitation wavelengths, respectively, were attributed to the PET and PVC plastic types. PVC has a higher fluorescence intensity at an excitation wavelength of 488 nm than PET. The plastic types PC, PE, PP, and PS showed nearly no fluorescence intensity at 405 nm and 488 nm excitation. In addition to the graphical representation of the fluorescence spectra, the obtained fluorescence maxima and Stokes shifts are listed in Table S1 (SI section C.1.) for the three excitation wavelengths: No fluorescence maxima were found for PC, PP, and PS at 405 nm excitation, which could be due to a lower excitation power and a lower quantum efficiency of the mini-spectrometer in this wavelength region. At 445 nm and 488 nm excitation, the fluorescence maxima, corresponding wavelengths, and Stokes shifts were determined for each material, showing no characteristics to identify them. By comparing our results with those of Gies et al. [23], where researchers were able to differentiate between plastic types and environmental materials using an excitation wavelength of 266 nm, we found that differentiation was not possible at our chosen excitation wavelengths using the fluorescence maxima or Stokes shifts, as no characteristics were found (Table S1, SI section C.1.).

Comparing the fluorescence spectra of the different plastic types, natural materials from terrestrial environments, and tire wear particles, grass can be differentiated due to the high chlorophyll fluorescence in the 600–700 nm region. The wood types show high fluorescence signals and a higher Stokes shift than the plastic at every excitation wavelength. Humus, clay, and sand show no fluorescence signal when excited with 405 nm excitation wavelength and exhibit a broad fluorescence emission when excited with 445 nm or 488 nm.

In conclusion, 405 nm might seem superior for the FD-FLIM measurements, as some natural materials have shown no fluorescence. However, PC, PP, and PS cannot be detected. Furthermore, plastics cannot be discriminated by the obtained fluorescence spectra independently of the wavelength. The highest fluorescence intensities are detected at an excitation wavelength of 445 nm due to a higher excitation power (light source), transmission (setup), and quantum efficiency (detector), which is advantageous for FD-FLIM measurements. Additionally, grass can be differentiated from other materials due to the high chlorophyll fluorescence at longer wavelengths. Thus, no statement of a superior wavelength can be given only by the fluorescence spectroscopy results; hence, all the granules are measured using the three excitation wavelengths.

### FD-FLIM

Using the described experimental setup and procedure and the introduced evaluation algorithm for FD-FLIM images, the data are graphically represented in diagrams, and the fluorescence lifetimes are calculated. The diagrams represent the normalized relative frequency of the taken phase- and modulation-dependent fluorescence lifetime images at 405 nm, 445 nm, and 488 nm excitation and are displayed in Figure S3 (SI section C.2.). In addition to the graphical evaluation shown in Figure S3 (SI section C.2.), the calculated expectation values and standard deviations assuming a Gaussian normal distribution of the phase- and modulation-dependent fluorescence lifetimes from plastic types are shown in Table S2 (SI section C.2.), whereby it can be derived that the fluorescence lifetimes increase at higher fluorescence excitation wavelengths. This behavior was already found in [24] but has yet to be further described. However, a difference in fluorescence lifetimes occurs because technical polymers cannot be counted as single fluorophores, which would exhibit equal fluorescence lifetimes independent from excitation. Additionally, as we measure the fluorescence lifetime of a single emission state and the entire fluorescence spectra, each fluoresce substance from the spectra contributes to an intensity-weighted fluorescence [25]. In this context, we argue that the difference in fluorescence lifetimes, i.e., the increasing fluorescence lifetimes at higher excitation wavelengths, is caused by a difference in the fluorescence intensity of the single substances.

The conclusion for the graphical and computational evaluation of 405 nm excitation is that some plastic types can be distinguished and identified from others. However, there is too much overlap in some cases, making identification difficult. Excitation at 445 nm revealed that all plastic types can be distinguished and unambiguously identified within one standard deviation based on their phase- and modulation-dependent fluorescence decay times, except for PS and PP. The calculated phase- and modulation-dependent fluorescence lifetimes from the 488 nm excitation showed that PVC and ABS can be identified utilizing their phase-dependent fluorescence lifetimes, and partial differentiation of the other plastic types is possible using phase- and modulation-dependent fluorescence lifetimes.

**Fig. 4:**
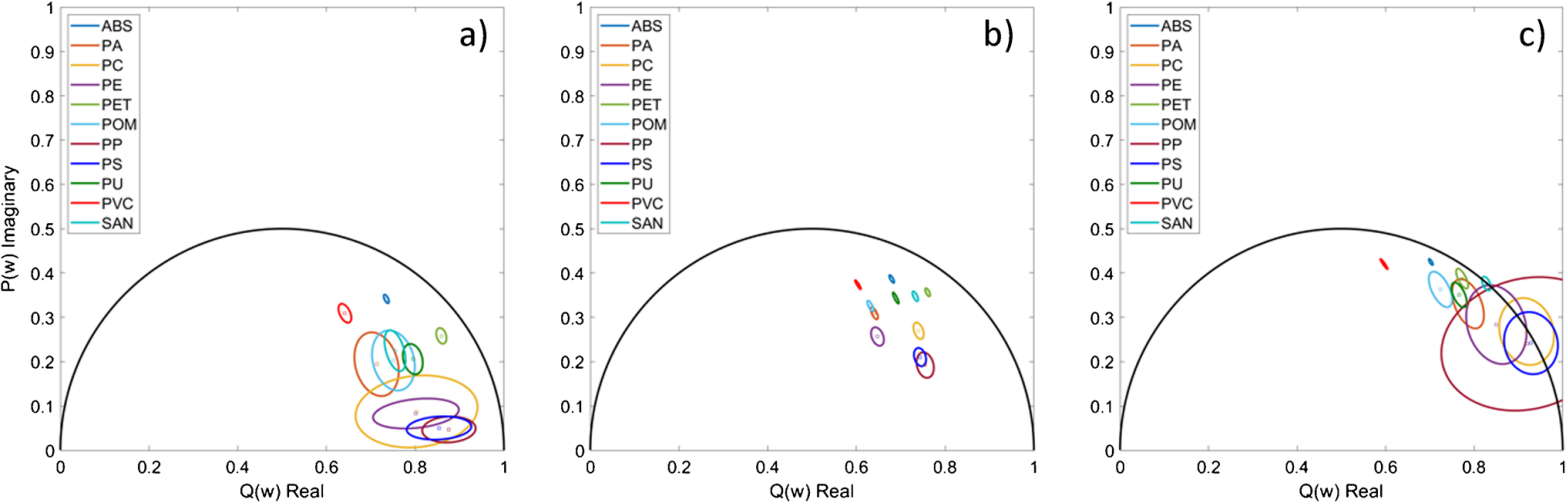
Phasor plots of the eleven different plastic types at 405 nm in **a**, 445 nm in **b**, and 488 nm excitation in **c**. Here, the square in the middle of the ellipse is the calculated expectation value of the 50 measurements per plastic type, and the ellipse represents the standard deviation of the modulation index and phase shift of the 50 measurements

The phasor plots, which represent a combination of the phase and modulation lifetime of each excitation wavelength, are displayed in Fig. 4. The phasor plot of the 405 nm excitation wavelength clearly shows that ABS (blue) and PVC (red) can be distinguished. In addition, the other plastic types can be differentiated, except for the combinations of SAN and POM, as well as PC, PE, PP, and PS. Due to the high chance of false identification of six plastic types, the probability calculation was omitted in the case of 405 nm excitation. The phasor plot of 445 nm in Fig. 4 b allows the identification and differentiation of each plastic type with an accuracy of 100%, except for differentiating PP from PS and POM from PA, as these plastic types show slightly overlapping ellipses. Using Eq. (4), the probabilities that PP can be differentiated from PS and vice versa are 70% (PP from PS) and 39% (PS from PP), respectively. The probabilities of differentiating PA from POM are 80% (PA from POM) and 72% (POM from PA), respectively. With 488 nm excitation (Fig. 4c), the phasor plot reveals that ABS, PA, POM, SAN, and PET can be identified and distinguished from the other plastic types. Differentiating the combinations of PU and PA, as well as PC, PE, PP, and PS, is impossible using the phasor plot in Fig. 4c. Thus, the probability calculation was also omitted in the case of 488 nm excitation.

These findings are in accordance with the findings of Langhals et al., Monteleone et al., and Gies et al. [20, 23, 24], who showed that plastics can be identified using TD fluorescence lifetime measurements. Additionally, the phasor plot representation was employed for successfully differentiating between different plastic types, per Monteleone et al. [35], where a phasor plot was created from TD measurements and used to differentiate plastic types. Nevertheless, a comparison of the measured fluorescence lifetimes is not possible, as TD fluorescence lifetime measurements were done at one single emission wavelength, and we measured the fluorescence lifetime of the whole fluorescence spectrum, where different substances proportionately contribute to convoluted phase-dependent and modulation-dependent fluorescence lifetimes.

To conclude, according to the investigations of 50 measurements of each plastic type using three different excitation wavelengths, the 445 nm excitation wavelength yields the best results. The three evaluation methods can identify and distinguish all the plastic types. Combining the three evaluation methods at 405 nm and 488 nm excitation makes differentiating some plastic types possible. However, PP, PC, PS, and PP cannot be distinguished because of their low fluorescence intensity and, thus, high standard deviation and noisy fluorescence lifetime images. As the results are best at 445 nm, only fluorescence lifetime images at 445 nm were taken to investigate the differentiability of natural materials from terrestrial environments and plastic types.

### Differentiation between plastics and natural materials from terrestrial environments via FD-FLIM

The results of the graphical identification and differentiation of the eleven plastic types from ten natural materials from terrestrial environments and tire wear particles using normalized histograms are shown in Figure S4 (SI section C.3.). Additionally, the calculated expectation values and standard deviations of the phase- and modulation-dependent fluorescence lifetimes resulting from the Gaussian evaluation are shown in Table S3 (SI section C.3.).

**Fig. 5:**
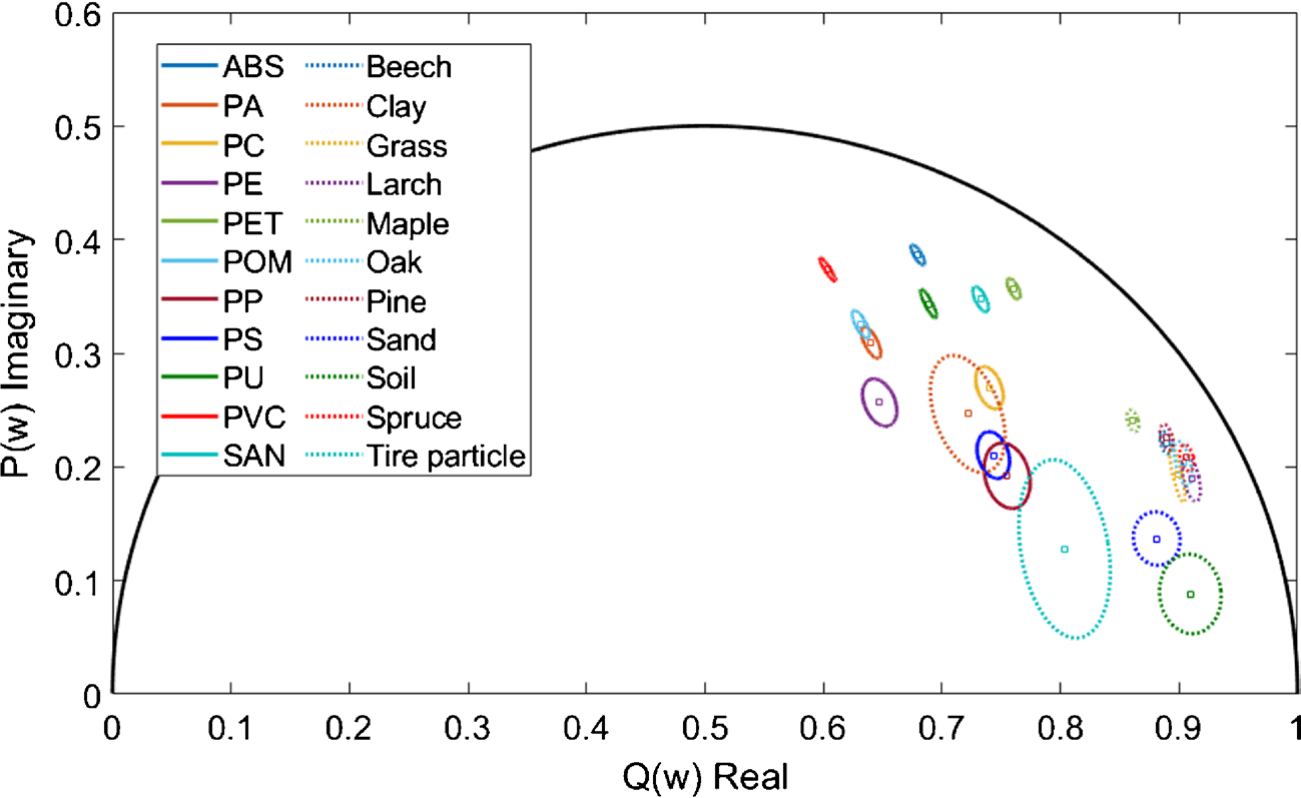
Phasor plot of the eleven different plastic types, ten terrestrial natural materials from terrestrial environments, and tire wear particles at 445 nm excitation

The phase-dependent and modulation-dependent fluorescence lifetimes of the 22 materials ranged from 0 to 3.5 ns and 1 to 6.5 ns, respectively. The phasor plot in Fig. 5 shows that all the materials characterized and their standard deviations (ellipses) lie well apart except for clay, PC, PP, PS, and tire wear material. According to Eq. (4), we calculated the probability of differentiating the materials clay/PC, clay/PP, clay/PS, and PP/tire wear material as 92% (clay from PC), 43% (PC from clay), 94% (clay from PP), 84% (PP from clay), 87% (clay from PS), 29% (PS from clay), 95% (PP from tire wear material), and 99% (tire wear material from PP). Thus, with a statistical significance of 95%, the PP and tire wear material can be unambiguously identified. In the other three cases, more measurements of the material must be taken to differentiate these or other statistical features, which must be taken into the evaluation and differentiation process. Thus, plastics, natural materials from terrestrial environments, and tire wear material can unambiguously be identified and distinguished using FD-FLIM with a high probability employing the phasor plot representation. Furthermore, the calculated fluorescence lifetimes of the natural materials from terrestrial environments and tire wear materials from Table S3 (SI section C.3.) and plastic types from Table S2 (SI section C.2.) support the hypothesis that plastics can be identified and distinguished from natural materials from terrestrial environments by employing their phase- and modulation-dependent fluorescence lifetimes. Furthermore, the measured phase- and modulation-dependent fluorescence lifetimes could be used for further investigations as a database for developing a classification system.

Although the investigated samples and the technique for TD and FD fluorescence lifetime measurements and thus the measured lifetimes are different, our results are in agreement with the findings of Gies et al. [23], where plastic types and mainly aquatic environmental materials were discriminated using fluorescence lifetime measured in TD: It is possible to distinguish between plastic types and environmental materials utilizing the material-specific fluorescence lifetime measured in TD or FD. However, we assume that identification via FD-FLIM is possibly much faster (depending on the polymer type, FD: maximum measurement time 16 s; TD: up to several minutes) and equally reliable (all polymers and environmental materials differentiated) as TD fluorescence lifetime measurements are.

## Conclusion

At present, there is a need to develop a rapid and simultaneously reliable method for analyzing MP in environmental samples that records essential particle properties such as shape and size for assessing the level of contamination and forming the basis for a reliable ecological risk assessment.

As currently used mass-based and particle-based chemical identification methods have certain disadvantages, developing a method for identifying MPs directly in environmental matrices or at least a method that allows for the timesaving reduction of purification steps compared to spectroscopic techniques is necessary. In this context, we aimed to optimize FD-FLIM measurement parameters for plastic types and natural materials from terrestrial environments to establish a basic analytical framework for directly identifying microplastics in low-complexity terrestrial environmental matrices. Therefore, we investigated (I) if there is a general optimum wavelength for identifying different plastic types simultaneously and (II) whether plastic types and natural materials from terrestrial environments can be identified and differentiated at one superior wavelength.

To answer (I), we measured and evaluated the fluorescence spectra and fluorescence lifetime of 22 materials, showing that 445 nm is superior for our experimental setup. Using the 445 nm excitation wavelength, we identified and differentiated the plastic types, tire wear particles, and natural materials from terrestrial environments via FD-FLIM, which answers our (II) question. Combining these results with the possibility of precisely determining plastic particles’ size, shape, and morphology during FD-FLIM measurements [26, 28] potentially allows for rapid assessment of the contamination level of environments.

However, further challenges arise from the presented results. First, as our experiments were conducted with technical plastic granules, the influence of fillers, additives, and dyes on the fluorescence properties, especially the fluorescence lifetimes of different plastic types, should be studied more thoroughly. In this context, only a few studies exist that show logarithmic dependencies of the fluorescence lifetime on filler or dye concentrations (e.g., see [30] and [36]). Second, further studies should cover environmentally coated MPs [37] to determine how a potentially strong fluorescent biofilm influences the fluorescence lifetime of plastic particles and, thus, the identifiability via FD-FLIM. In this context, the next step would be to establish an extensive FD-FLIM database on plastic materials with different properties and additional environmental materials through which plastic particles could be classified directly in an environmental matrix and, if possible, even on-site. These examples show that several open questions must be addressed before FD-FLIM can routinely analyze MP samples.

However, our results demonstrated the high potential of the FD-FLIM method for identifying plastic types and distinguishing them from natural materials from terrestrial environments with high probability. We showed that FD-FLIM is promising, especially for MP analysis in samples of relatively low complexity, such as a surface inspection of agricultural farmland soil, which is possible and may be used for on-site MP identification in various ecosystems after further development.

### Supplementary Information

Below is the link to the electronic supplementary material.

Supplementary file1 (DOCX 1190 KB)
