# Supplementary material for: Identification of different plastic types and natural materials from terrestrial environments using fluorescence lifetime imaging microscopy
Source: Anal Bioanal Chem. 2024 Apr 23;416(15):3543–54. doi: 10.1007/s00216-024-05305-w (PMC11156735; doi:10.1007/s00216-024-05305-w)
Supplement: Supplementary file 1 — Supplementary file1 (DOCX 1190 KB) [file 216_2024_5305_MOESM1_ESM.docx]

Cover Sheet: Supporting Information

Manuscript: **Identification of different plastic types and natural materials from terrestrial environments using fluorescence lifetime imaging microscopy**

Maximilian Wohlschläger^1^, Martin Versen^1^, Martin G.J. Löder^2^, Christian Laforsch^2^

^1^ Faculty of Engineering, Technical University of Applied Sciences Rosenheim, Hochschulstraße 1, 83024 Rosenheim, Germany

^2^ Animal Ecology I and BayCEER, University Bayreuth, Universitätsstraße 30, 95440 Bayreuth, Germany.

The supporting information consists of additional information on the FD-FLIM technique (**SI section B.1. Theory**), additional information on the experimental setup (**SI section B.2. Experimental setup for the spectral analysis and measurement of the fluorescence lifetime of plastics and microplastics**) a more informative description of the results (**SI section C.1.: Fluorescence spectroscopy; SI section C.2.: FD-FLIM; SI section C.3.: Differentiation between plastics and natural materials from terrestrial environments via FD-FLIM).** Three Figures are included in the SI: Figure S1 shows the experimental setup schematically represented; Figure S2 shows the normalized histograms of the determined phase- and modulation-dependent fluorescence lifetimes; Figure S3 shows the normalized histograms of the phase- and modulation-dependent fluorescence lifetimes at an excitation wavelength of 445 nm from the eleven plastic types, tire material, and ten natural materials from terrestrial environments. The calculated spectral parameters ($I_{max}$ [a.u.]; $\lambda_{max}$ [nm]; $\Delta\lambda$ [nm]) are shown in Table S1. Furthermore, the calculated phase- and modulation-dependent fluorescence lifetimes of the plastic types at different excitation wavelengths and of the natural materials at 445 nm excitation are entered in Tables S2 and S3. In total, there are fourteen pages (S1-S14).

**SI section B.1. Theory: Explanation of the camera technique to measure FD-FLIM:**

An FD-FLIM camera measures the fluorescence intensity at 16 equidistant defined times. Therefore, two taps (tap A and tap B) are installed in one pixel of the FD-FLIM camera. Tap A integrates the fluorescence photons over the first half of the oscillation, i.e., between 0 and 180 degrees, in the first step. After 180 degrees are reached, a switch in the pixel is toggled, and tap B is activated, which integrates the photons between 180 degrees and 360 degrees. The two-photon integrations in taps A and B result in fluorescence intensities I1 and I9. In step 2), the starting angle where tap A is active is shifted by 22.5 degrees so that the photon integration starts at 22.5 degrees and ends at 202.5 degrees. When 202.5 degrees are reached, the switch is toggled, and the fluorescence photons are integrated into tap B from 202.5 degrees to 382.5 degrees. The integration process and the shifting of the starting angle by +22.5 degrees are repeated eight times, resulting in 16 pixel-dependent fluorescence intensities. As the fluorescence intensities are measured simultaneously for each pixel, 16 fluorescence intensity images are created. Reconstruction of the fluorescence oscillation is possible using the 16 measured fluorescence intensity images.

**SI section B.2. Experimental setup for the spectral analysis and measurement of the fluorescence lifetime of plastics and microplastics**

To investigate whether plastic types can be identified utilizing the areal fluorescence lifetime measured in the frequency domain, an experimental setup consisting of laser diodes (LaserNest 405-300, LaserNest 445-500, and PhoxX+ 488-200) from Omicron-Laserage GmbH, an FD-FLIM camera system pco.flim from Excelitas PCO GmbH, a probe station (EPS150FA from Cascade Microtech) including a microscope (PSM-1000 from Motic) and optical filters was used (see Figure S1). The FD-FLIM camera can also be exchanged for a spectrometer (Minispectrometer from Hamamatsu) to measure the fluorescence spectrum. As one of the primary goals of these investigations is to define the excitation wavelength at which plastic types can be reliably identified and distinguished, three laser diodes with different excitation wavelengths of 405 nm, 445 nm, and 488 nm were used. The spectral fluorescence was measured in the 280 to 1000 nm range, and the spectral resolution was 1 nm. An areal measurement of 1004x1008 fluorescence lifetimes from 100 ps to 100 µs with a dynamic range of 10 bits is possible using the pco.flim. A single pixel measures the location-dependent fluorescence intensity, the phase shift, and the modulation index. Using the measured phase shift and modulation index, phase-dependent and modulation-dependent fluorescence lifetime images were calculated according to the described theory (see equations (1) and (3)). The microscope provides magnifications of x2, x10, and x20, whereby a magnification of x20 is used for the investigations. The magnification causes the image section of the camera to cover an area of 0.28 mm × 0.28 mm (pixel size: 0.28 µm × 0.28 µm). Since three different laser diodes are used during the investigations, two optical filters are selected for each wavelength. The first optical filter is a laser clean-up filter used to narrow the bandwidth of the excitation light in the excitation light path of the microscope (exciter). The second is a long-pass (LP) filter to block reflected and stray light from the plastic sample. When laser diodes with excitation wavelengths of 405 nm, 445 nm, or 480 nm are used, the corresponding LP filters have cut-on wavelengths of 420 nm, 460 nm, or 500 nm, respectively.


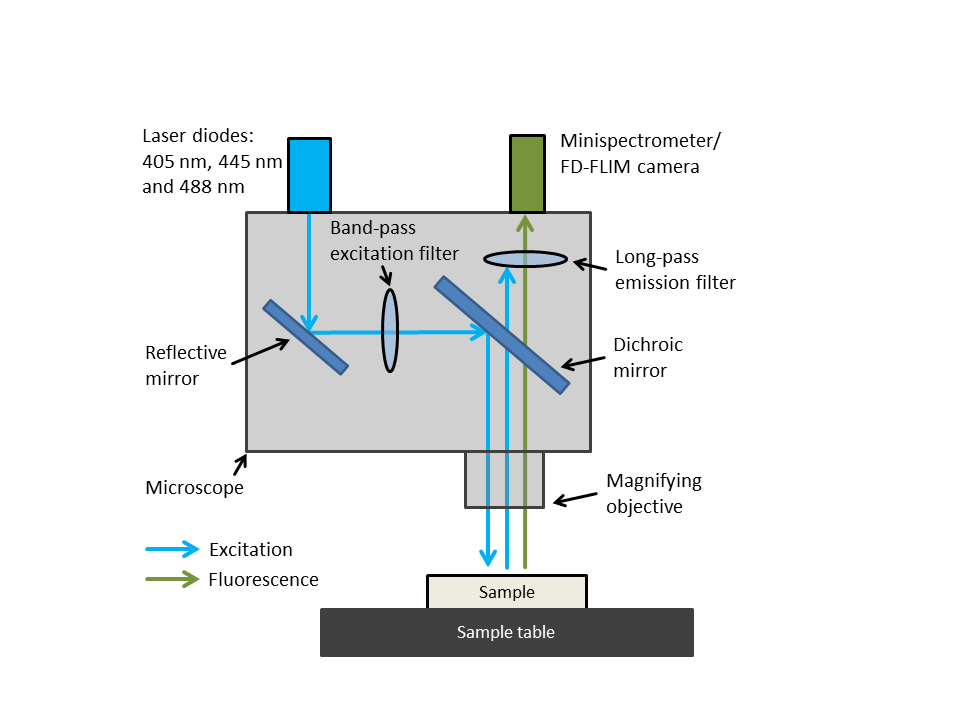


Figure S1. Schematic representation of the experimental setup consisting of laser diodes, a minispectrometer/FD-FLIM camera, an optical band-pass excitation filter, and an optical long-pass emission filter assembled on the microscope.

**SI section C.1. Fluorescence spectroscopy**

Table S1. Results of the spectral fluorescence measurements of the eleven different plastic types at 405 nm, 445 nm, and 488 nm excitation.

| Excitation wavelength 405 nm | | | |
| --- | --- | --- | --- |
| Material | $I_{max}$ **[a.u.]** | $\lambda_{max}$ **[nm]** | $\Delta\lambda$ **[nm]** |
| ABS | 3912 | 444 | 39 |
| PA | 114 | 445 | 40 |
| PC | -- | -- | -- |
| PE | 43 | 459 | 54 |
| PET | 697 | 444 | 39 |
| POM | 100 | 447 | 42 |
| PP | -- | -- | -- |
| PS | -- | -- | -- |
| PU | 208 | 443 | 38 |
| PVC | 343 | 446 | 41 |
| SAN | 171 | 443 | 38 |
| Beech | 353 | 542 | 137 |
| Clay | -- | -- | -- |
| Grass | 4152 | 688 | 283 |
| Larch | 283 | 542 | 137 |
| Maple | 1051 | 520 | 115 |
| Oak | 393 | 530 | 125 |
| Pine | 521 | 532 | 127 |
| Sand | -- | -- | -- |
| Soil | -- | -- | -- |
| Spruce | 679 | 519 | 114 |
| Tire | 61 | 443 | 38 |
| Excitation wavelength 445 nm | | | |
| ABS | 22871 | 519 | 74 |
| PA | 437 | 518 | 73 |
| PC | 293 | 524 | 79 |
| PE | 376 | 514 | 69 |
| PET | 4260 | 521 | 76 |
| POM | 652 | 520 | 75 |
| PP | 229 | 514 | 69 |
| PS | 269 | 518 | 73 |
| PU | 1403 | 545 | 100 |
| PVC | 4757 | 528 | 83 |
| SAN | 1032 | 532 | 87 |
| Beech | 21128 | 553 | 108 |
| Clay | 360 | 546 | 101 |
| Grass | 31787 | 733 | 288 |
| Larch | 13081 | 556 | 111 |
| Maple | 18419 | 552 | 107 |
| Oak | 15743 | 553 | 108 |
| Pine | 18816 | 559 | 114 |
| Sand | 568 | 545 | 100 |
| Soil | 266 | 556 | 111 |
| Spruce | 26004 | 552 | 107 |
| Tire | 159 | 536 | 91 |
| Excitation wavelength 488 nm | | | |
| ABS | 12944 | 536 | 48 |
| PA | 165 | 556 | 68 |
| PC | 109 | 538 | 50 |
| PE | 103 | 560 | 72 |
| PET | 1015 | 546 | 58 |
| POM | 267 | 557 | 69 |
| PP | 51 | 566 | 78 |
| PS | 63 | 536 | 48 |
| PU | 340 | 558 | 70 |
| PVC | 2208 | 548 | 60 |
| SAN | 863 | 601 | 113 |
| Beech | 7336 | 561 | 73 |
| Clay | 174 | 576 | 88 |
| Grass | 45631 | 691 | 203 |
| Larch | 7673 | 570 | 82 |
| Maple | 9001 | 562 | 74 |
| Oak | 6469 | 564 | 76 |
| Pine | 10737 | 582 | 94 |
| Sand | 235 | 566 | 78 |
| Soil | 151 | 573 | 85 |
| Spruce | 9048 | 560 | 72 |
| Tire | 64 | 534 | 46 |

**SI section C.2. FD-FLIM**

405 nm excitation wavelength:

At an excitation wavelength of 405 nm, the measured phase-dependent fluorescence lifetimes are in the range of 0 ns – 3 ns, and the modulation-dependent fluorescence lifetimes range from 0 ns – 8 ns. ABS and PVC are not differentiable from each other in the graphical histogram representation but can be distinguished from the other plastic types in Figure S2 a). Moreover, PA, PET, POM, PU, and SAN cannot be differentiated using Figure S2 a) and b) but can be distinguished from the other plastic types. Two- and three-phase-dependent fluorescence lifetimes can be obtained for the plastic type PP and PS, respectively. In contrast to PCs, which exhibit two modulation-dependent fluorescence decay times, PP and PS exhibit only one modulation-dependent fluorescence lifetime. The results of multiple determined fluorescence lifetimes for PP, PS, and PC complicate the differentiation of the three plastic types. However, PP, PS, and PC can clearly be distinguished from the other plastic types.

Furthermore, the fluorescence spectra showed that PP, PS, and PC have nearly no fluorescence intensity when excited at 405 nm, which led to the conclusion that the fluorescence lifetime images are noisy; thus, more than one fluorescence lifetime was found for those plastic types. The low fluorescence intensity also causes a higher standard deviation in the Gaussian evaluation algorithm, leading to overlapping of the Gaussian curves and complicating the differentiation of the other plastic types even further. In conclusion, the histogram plot of the FD-FLIM measurements at an excitation wavelength of 405 nm shows that no clear distinction between the plastic types is possible, neither using the phase nor the modulation-dependent fluorescence lifetime.

445 nm excitation wavelength:

In Figure S2 c) and d), the normalized relative frequencies of the measured phase- and modulation-dependent fluorescence lifetime images are illustrated at an excitation wavelength of 445 nm. From the normalized histograms in Figure S2 c) and d), each plastic type can obtain one characteristic phase- and modulation-dependent fluorescence lifetime. Here, the phase-dependent fluorescence lifetimes range from 0.5 ns to 3.5 ns, and the modulation-dependent fluorescence lifetimes range from 3 ns to 6 ns. Additionally, all plastic types, except for the combinations of PP and PS with PET, POM, and SAN, can be differentiated within one standard deviation by the phase-dependent fluorescence lifetime histogram. The modulation-dependent fluorescence lifetime histogram can distinguish PET, POM, and SAN. Thus, differentiation and identification of all plastic types are possible using normalized relative frequency diagrams of the phase- and modulation-dependent fluorescence lifetimes, except for those of PP and PS. In addition, the variance in the single plastic types' phase- and modulation-dependent fluorescence lifetimes is lower than that in the 405 nm fluorescence lifetime histograms in Figure S2 a) and b). The reason is that higher fluorescence intensities (see Figure S2 c) and d) lead to more minor standard deviations; thus, differentiating between the plastic types is easier.

488 nm excitation wavelength:

Figure S2 e) and f) show the phase- and modulation-dependent fluorescence lifetimes measured at 488 nm excitation. The normalized frequency histograms in Figure S2 e) and f) show that the phase-dependent fluorescence lifetimes range from 0 ns to 4.3 ns, and the modulation-dependent fluorescence lifetime lies from 0 ns to 6 ns. Figure S2 e) shows that every plastic type has a single-phase-dependent fluorescence lifetime. However, PA, PC, PE, PP, and PS have more than one modulation-dependent fluorescence lifetime, according to Figure S2 f). Using Figure S2 e), the plastic types of PVC and ABS can be distinguished from the other plastics. Differentiating the other plastic types is difficult using the phase-dependent fluorescence lifetime histogram Figure S2 e) and the modulation-dependent fluorescence lifetime Figure S2 f). As already described in the Results section for the 405 nm excitation wavelength, the low fluorescence intensity of some plastic types aggravates distinguishing these plastics' fluorescence lifetime signals in the histograms when excited at 488 nm.

In conclusion, a graphical evaluation of the normalized relative frequency of the phase- and modulation-dependent fluorescence lifetimes at different excitation wavelengths showed that the best result was obtained at an excitation wavelength of 445 nm. Additionally, the fluorescence intensity strongly influences detecting a representative signal to measure the fluorescence lifetime.


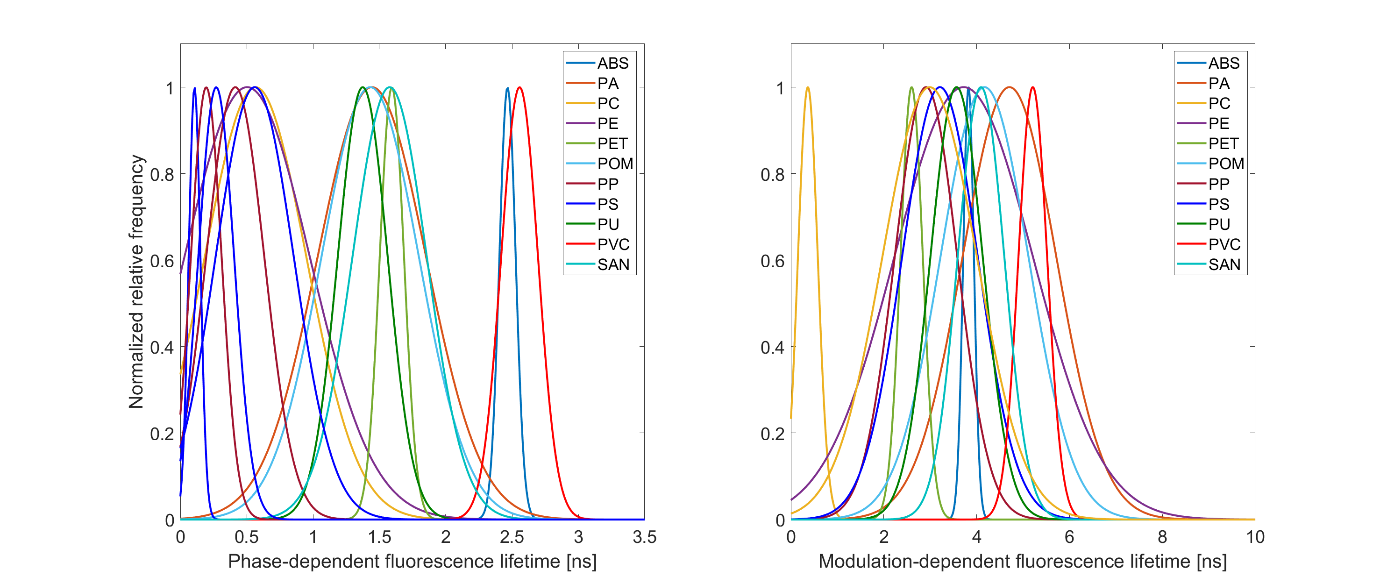

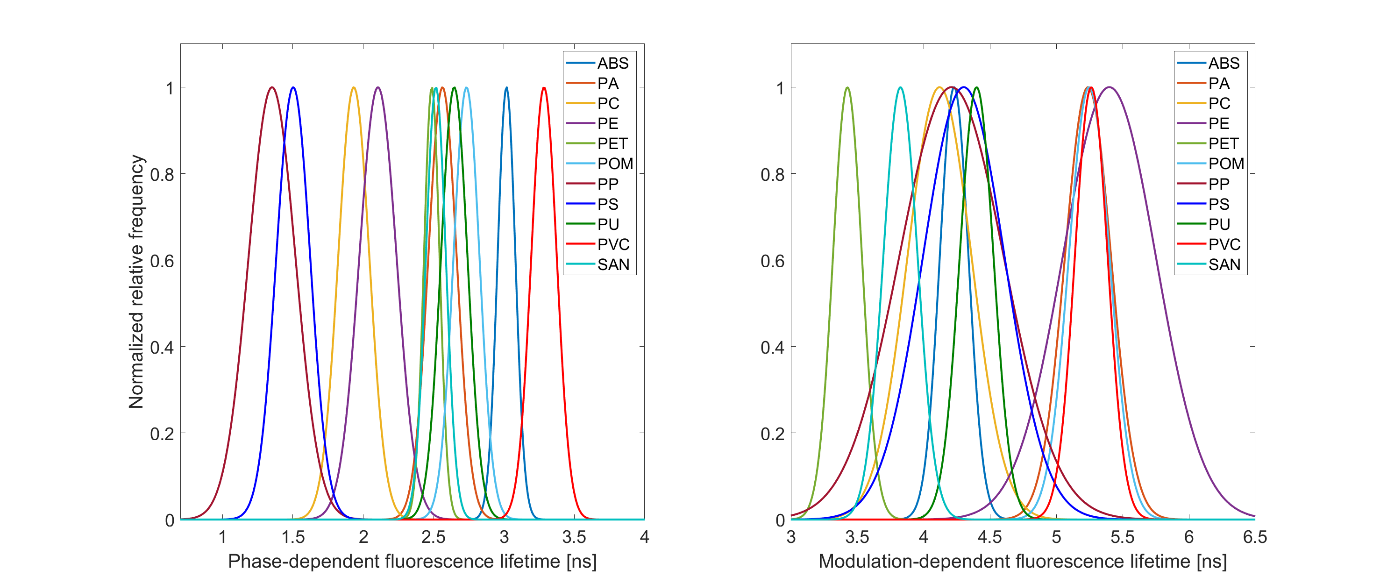

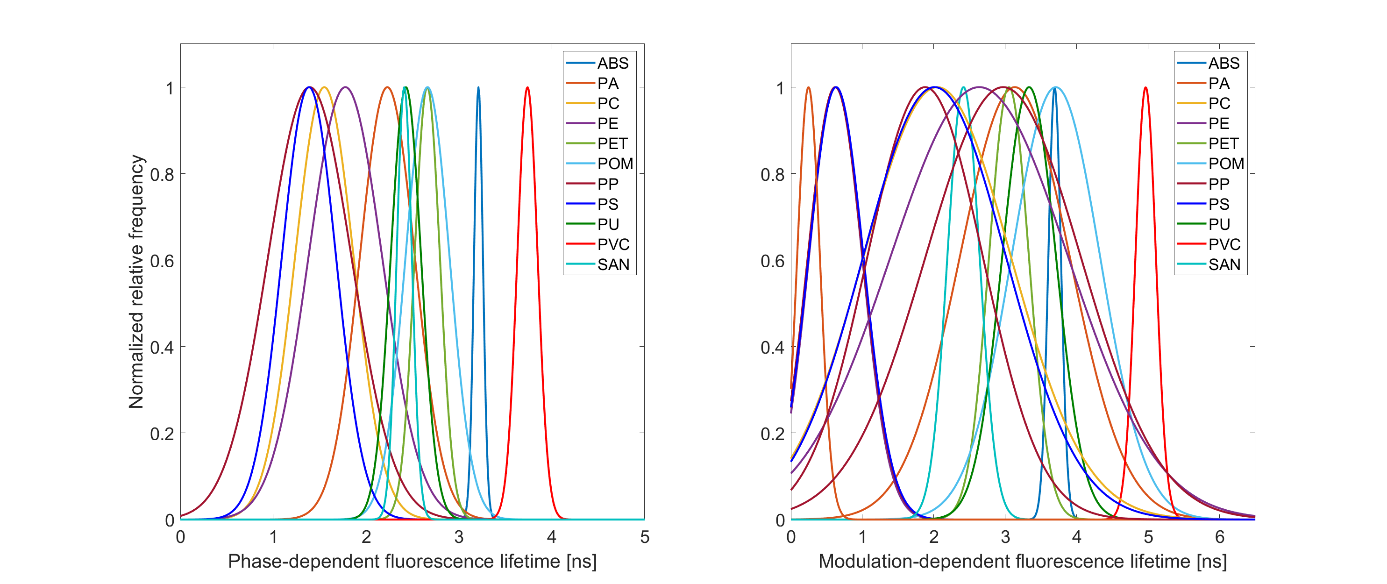


a)

b)

d)

c)

f)

e)

Figure S2. Normalized relative frequency as a function of the phase-dependent fluorescence lifetime (left) and modulation-dependent fluorescence lifetime (right) of the eleven different plastic types with excitation wavelengths of 405 nm in a) and b), 445 nm in c) and d) and 488 nm in e) and f).

Table S2. Calculated expectation values and standard deviations assuming a Gaussian normal distribution from the phase-dependent and modulation-dependent fluorescence lifetimes of the eleven plastic types at 405 nm, 445 nm, and 488 nm excitation.

| Excitation wavelength 405 nm | | |
| --- | --- | --- |
| Plastic-type | $\tau_{\phi i}$ [ns] | $\tau_{Mi}$ [ns] |
| ABS | 2.47 ± 0.06 | 3.83 ± 0.12 |
| PA | 1.44 ± 0.41 | 4.71 ± 1.01 |
| PC | 0.57 ± 0.38 | 0.37 ± 0.21  2.99 ± 1.02 |
| PE | 0.50 ± 0.47 | 3.72 ± 1.49 |
| PET | 1.59 ± 0.09 | 2.60 ± 0.24 |
| POM | 1.43 ± 0.37 | 4.17 ± 0.95 |
| PP | 0.41 ± 0.22  0.20 ± 0.12 | 2.91 ± 0.68 |
| PS | 0.11 ± 0.04  0.27 ± 0.14  0.56 ± 0.30 | 3.21 ± 0.82 |
| PU | 1.37 ± 0.19 | 3.57 ± 0.56 |
| PVC | 2.56 ± 0.14 | 5.21 ± 0.31 |
| SAN | 1.58 ± 0.28 | 4.09 ± 0.50 |
| Excitation wavelength 445 nm | | |
| ABS | 3.02 ± 0.06 | 4.23 ± 0.11 |
| PA | 2.56 ± 0.10 | 5.24 ± 0.17 |
| PC | 1.93 ± 0.11 | 4.12 ± 0.24 |
| PE | 2.10 ± 0.13 | 5.40 ± 0.35 |
| PET | 2.49 ± 0.06 | 3.43 ± 0.11 |
| POM | 2.73 ± 0.09 | 5.25 ± 0.15 |
| PP | 1.35 ± 0.17 | 4.21 ± 0.39 |
| PS | 1.50 ± 0.12 | 4.30 ± 0.31 |
| PU | 2.65 ± 0.09 | 4.40 ± 0.13 |
| PVC | 3.28 ± 0.09 | 5.26 ± 0.12 |
| SAN | 2.52 ± 0.07 | 3.83 ± 0.13 |
| Excitation wavelength 488 nm | | |
| ABS | 3.21 ± 0.05 | 3.69 ± 0.09 |
| PA | 2.23 ± 0 30 | 3.13 ± 0.77  0.25 ± 0.16 |
| PC | 1.55 ± 0.32 | 0.63 ± 0.38  2.05 ± 1.03 |
| PE | 1.78 ± 0.40 | 0.62 ± 0.37  2.64 ± 1.25 |
| PET | 2.65 ± 0.14 | 3.05 ± 0.28 |
| POM | 2.66 ± 0.24 | 3.71 ± 0.62 |
| PP | 1.39 ± 0.45 | 0.62 ± 0.39  1.88 ± 0.81  2.97 ± 1.09 |
| PS | 1.38 ± 0.29 | 0.63 ± 0.39  2.01 ± 1.00 |
| PU | 2.43 ± 0.16 | 3.33 ± 0.38 |
| PVC | 3.74 ± 0.11 | 4.97 ± 0.14 |
| SAN | 2.41 ± 0.08 | 2.42 ± 0.23 |

**SI section C.3. Differentiation between plastics and natural materials from terrestrial environments via FD-FLIM**

From the phase-dependent fluorescence lifetime histograms (Figure S3 a)), it can be determined that the natural materials from terrestrial environments and plastic types differentiation is possible except for PC, PS, and PP. The phase-dependent fluorescence lifetimes of PC, PS, and PP superimpose the phase-dependent fluorescence lifetimes of beech, maple, oak, pine, and spruce, respectively, which does not allow differentiation of the three plastic types from the natural materials from terrestrial environments. Nevertheless, PC, PS, PP, and other plastic types can be distinguished from natural materials from terrestrial environments using modulation-dependent fluorescence lifetimes (see Figure S3 b)) except for clay and tire wear. Thus, identifying and differentiating plastic types is possible using both measured fluorescence lifetimes.

**
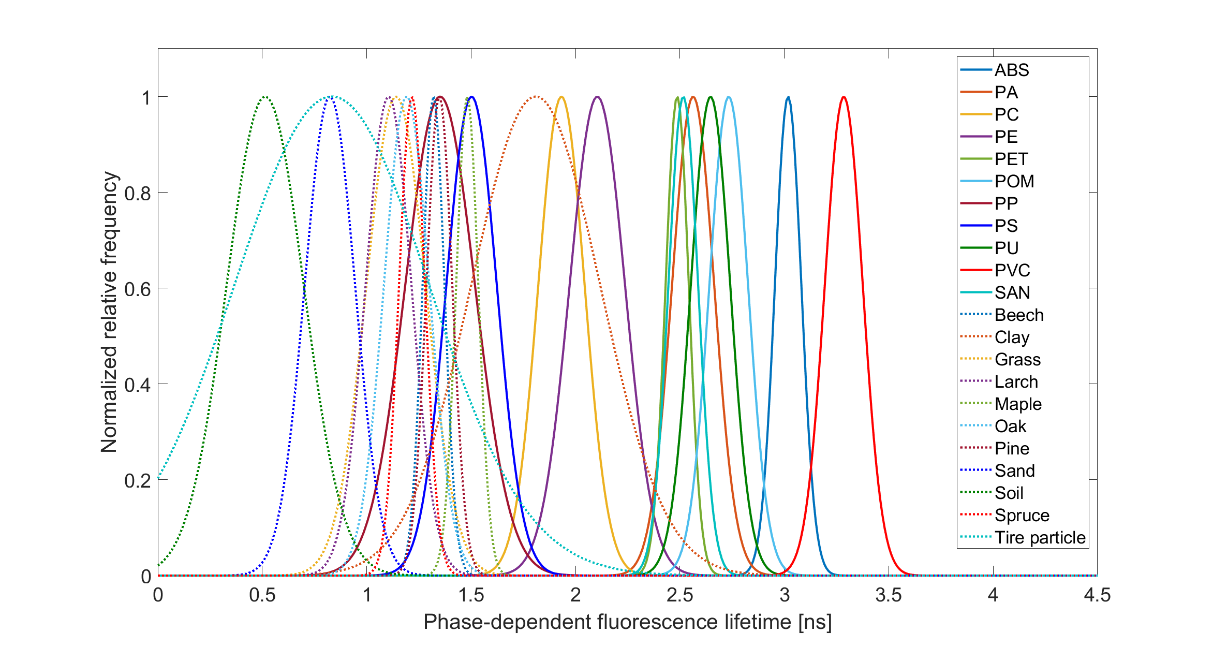

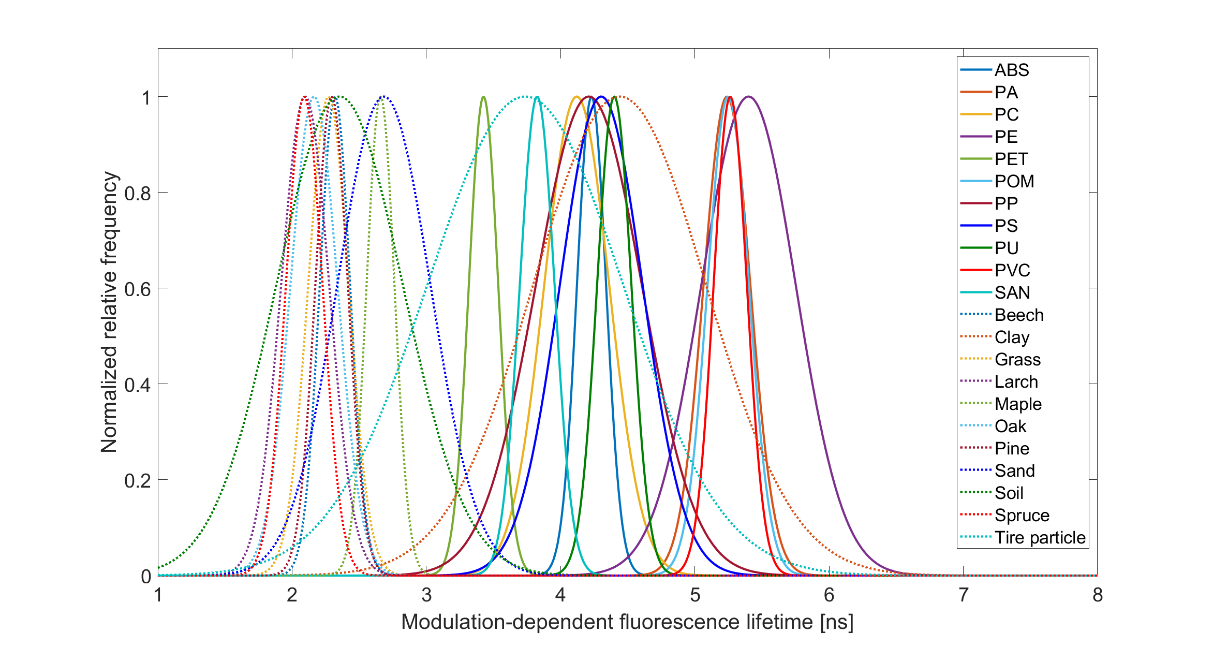
**

a)

b)

Figure S3. Normalized relative frequency as a function of the phase-dependent fluorescence lifetime in a) and as a function of the modulation-dependent fluorescence lifetime in b) of the eleven different plastic types and ten different natural materials from terrestrial environments as well as tire particles at 445 nm excitation.

Table S3. Calculated expectation values and standard deviations assuming a Gaussian normal distribution from the phase-dependent and modulation-dependent fluorescence lifetimes of the ten natural materials from terrestrial environments and tire material at 445 nm excitation.

| Excitation wavelength 445 nm | | |
| --- | --- | --- |
| Material | $\tau_{\phi i}$ [ns] | $\tau_{Mi}$ [ns] |
| Beech | 1.32 ± 0.05 | 2.32 + 0.11 |
| Clay | 1.81 ± 0.31 | 4.44 + 0.64 |
| Grass | 1.14 ± 0.14 | 2.27 + 0.15 |
| Larch | 1.10 ± 0.11 | 2.10 + 0.18 |
| Maple | 1.48 ± 0.05 | 2.66 + 0.10 |
| Oak | 1.19 ± 0.11 | 2.16 + 0.18 |
| Pine | 1.35 ± 0.06 | 2.29 + 0.12 |
| Sand | 0.82 ± 0.12 | 2.68 + 0.35 |
| Soil | 0.51 ± 0.18 | 2.35 + 0.47 |
| Spruce | 1.22 ± 0.06 | 2.09 + 0.14 |
| Tire | 0.83 ± 0.47 | 3.74 + 0.73 |
